# Disparities in attitudes toward field of study and future career among students at Yasuj University of medical sciences

**DOI:** 10.1186/s12909-025-08087-6

**Published:** 2025-12-20

**Authors:** Seyyed Masoud Moradian, Seyyed Amir Moradian, Zahra Amirkhani, Reza Namvar, Seyyede Nazaninzahra Gharib, Solaiman Afroughi

**Affiliations:** 1 Student Research Committee, Yasuj University of Medical Sciences, Yasuj, Iran; 2 Student Research Committee, Tabriz University of Medical Sciences, Tabriz, Iran; 3 Department of Nursing, School of Nursing, Larestan University of Medical Sciences, Larestan, Iran; 4 Student Research Committee, Larestan University of Medical Sciences, Larestan, Iran; 5 Department of Neuroscience and Cognition, Faculty of Advanced Medical Sciences, Tabriz University of Medical Sciences, Tabriz, Iran; 6 Department of Biology Education, Farhangian University, Shiraz, Iran; 7 Department of Biostatistics and Epidemiology, School of Health and Nutrition Science, Faculty of Health and Nutritional Sciences and Social Determinants of Health Research Centre, Yasuj University of Medical Sciences, Yasuj, Iran

**Keywords:** Attitude, Field of study, Career prospects, Medical students, Dental students, Paramedical education

## Abstract

**Background:**

Attitudes, shaped by cognitive and emotional factors, are fundamental constructs in social psychology that significantly influence academic and career success. These attitudes tend to evolve during the student years, a critical period for personal and professional development. Investigating students’ attitudes toward their academic fields and future careers offers valuable predictive insights into their future behavior. This study aimed to assess the attitudes of healthcare students at Yasuj University of Medical Sciences.

**Methods:**

In this cross-sectional study, 166 medical, dental, and paramedical students were surveyed using a validated 14-item questionnaire based on a 5-point Likert scale. Attitudes were classified into positive, neutral, and negative categories according to Bloom’s cut-off points. Data were analyzed using ANOVA and Pearson’s correlation coefficient, with statistical significance set at *p* < 0.05.

**Results:**

Dental students demonstrated the highest mean attitude scores toward their fields (15.74 ± 2.68), followed by nurse anesthetist and medical students, while operating room students had the lowest scores (10.91 ± 2.46). Item-level analyses revealed that 81.5% of dental students believed the income in their field was suitable, compared to just 12.0% in laboratory sciences. Additionally, 48.1% of dental students reported confidence in their career future, while agreement fell to 18.5% in radiology and 12.0% in laboratory sciences. Only dentistry and nurse anesthetist students showed over 40% agreement with the statement that employment opportunities were adequate, with rates as low as 8.0% in laboratory sciences. Over 90% of paramedical students reported neutral or negative attitudes toward their future careers, with the highest rates in operating room and laboratory sciences (100%). By contrast, medical and dental students showed more favorable outlooks, with 19.69% and 29.62% reporting positive attitudes, respectively. Weak but statistically significant correlations were found between student attitudes and academic term and gender, while no significant associations were observed with other demographic factors.

**Conclusions:**

The findings reveal systemic challenges within healthcare education, particularly among paramedical students, where neutral and negative attitudes predominate. These trends underscore the urgent need for policy reforms, enhanced career counseling services, and strategic workforce planning to foster student engagement, reduce professional disillusionment, and mitigate the broader impacts on the healthcare system.

**Supplementary Information:**

The online version contains supplementary material available at 10.1186/s12909-025-08087-6.

## Introduction

 Attitudes are pivotal constructs in social psychology, encapsulating the interplay of cognitive, emotional, and behavioral responses toward specific subjects [1]. In educational contexts, understanding students’ attitudes is crucial, as these orientations shape academic engagement, personal achievement, and long-term career trajectories [2]. Traditional psychological models emphasized the relative stability of attitudes as personality traits [3]; however, more recent research highlights their dynamic and context-dependent nature, particularly during pivotal transitions such as higher education [4, 5]. This perspective is substantiated by longitudinal studies conducted among medical students, which reveal that attitudes are subject to significant changes throughout training—often exhibiting a gradual decline over time as a result of diminished idealism, increased exposure to clinical realities, and the influence of the hidden curriculum [5].

Emerging evidence suggests that students’ attitudes evolve substantially in response to personal experiences, educational environments, and broader societal influences [6]. Transition periods, notably entry into university life, are recognized as significant catalysts for attitude development and change [5, 7]. Despite this recognition, the predictive role of evolving attitudes on academic engagement and career trajectories remains underexplored, particularly within healthcare education contexts.

Medical education, particularly in disciplines such as medicine, dentistry, and allied health sciences, presents a complex blend of academic rigor, clinical exposure, and professional socialization. These formative experiences can reinforce or erode students’ initial motivations and beliefs, thereby shaping their long-term professional identity [8]. Studies have shown that factors such as faculty support, curriculum structure, and perceived career opportunities significantly influence students’ attitudinal trajectories during training [9]. When these experiences are misaligned with students’ expectations or values, the resulting dissonance may contribute to disengagement, dissatisfaction, and reduced commitment to their chosen fields [10].

Moreover, attitudes formed during training do not only reflect individual perceptions but also mirror systemic realities such as institutional inequities, workforce saturation, and labor market instability [11]. In healthcare systems under strain—particularly in low- and middle-income countries—students may become acutely aware of professional challenges, including limited job prospects, income disparities, and the growing phenomenon of medical migration [12]. As such, educational institutions play a pivotal role not only in delivering academic content but also in shaping students’ psychological readiness and professional outlook through transparent communication, mentoring, and support systems [13]. Given the potential long-term implications of these attitudinal developments, further investigation into their determinants is both timely and necessary.

The present study investigates the attitudes of medical, dental, and paramedical students at Yasuj University of Medical Sciences toward their field of study, future education, and career. By analyzing these attitudes and their associations with demographic factors, the study seeks to contribute to the broader understanding of professional development challenges within Iran’s healthcare education system. Findings from this study are expected to inform future educational reforms and policy interventions aimed at enhancing professional motivation and retention in the healthcare workforce.

## Materials and methods

### Study design and setting

This cross-sectional, descriptive-analytical study was conducted during the 2024 academic year at Yasuj University of Medical Sciences in Iran. The study aimed to assess attitudes among students enrolled in medicine, dentistry, and bachelor’s degree paramedical programs (including medical laboratory science, radiology, nurse anesthetist, and operating room).

### Participants and sampling

The study population included all students who had completed at least one academic semester in their respective programs. Participants were selected through stratified random sampling, with academic field serving as the strata. Within each stratum, students were randomly selected using a random number generator to ensure proportional representation across disciplines.

The minimum required sample size (*n* = 166) was calculated using Cochran’s formula for finite populations, assuming a confidence level of 95% (α = 0.05), a conservative proportion estimate (*p* = 0.5), and a desired margin of error (d = 0.074). Students who submitted incomplete questionnaires were excluded from the final analysis.

This study was approved by the Ethics Committee of Yasuj University of Medical Sciences (IR.YUMS.REC.1403.007), and all participants provided informed consent electronically prior to participation.

### Instrument and measures

Data were collected using a validated 14-item questionnaire originally adapted from the University of Minnesota [14] and previously used by Samadi et al. [15]. The instrument aimed to assess students’ attitudes toward their field of study, future education, and career prospects.

Content validity was assessed by a panel of 10 experts in medical education, psychology, and health sciences using the Content Validity Index (CVI). Items with CVI scores ≥ 0.8 were retained. Following validation, the questionnaire was pilot-tested with 30 students from the target population, resulting in a Cronbach’s alpha of 0.836, indicating good internal consistency.

The final questionnaire included two parts: demographic information (gender, age, academic term, residence, marital status, family financial status, and parents’ education levels) and an attitude assessment section comprising 14 items rated on a 5-point Likert scale (1 = totally disagree to 5 = totally agree). Of the 14 attitude items, four questions assessed students’ perceptions of their field of study, while the remaining ten focused on their future education and career prospects. Attitude scores in each domain—field of study (4 items, total score range: 4–20) and future education/career (10 items, total score range: 10–50)—were converted into percentages, and categorized based on Bloom’s cut-off points [16]: ≥80% as positive, 60–79% as neutral, and < 60% as negative. This approach enabled a standardized classification of attitudes across scales with different item counts [17, 18]. The full questionnaire is provided in Supplementary Material 1.

### Data collection

Data collection was conducted online over a two-week period during the middle of the semester, intentionally avoiding examination periods and holidays to reduce response bias. Based on the official registrar’s roster, a stratified random sample of students was selected by academic field, and a unique survey link was sent directly to the institutional email addresses of the selected students. Each invitee received a standardized instruction sheet outlining the study objectives, procedures, confidentiality assurances, and voluntary nature of participation.

The questionnaire was administered via Google Forms, which permitted only one submission per invited student through institutional email authentication. All questions were mandatory, and respondents were able to review and revise their answers prior to final submission. The estimated completion time was approximately 8 to 10 min. Submitted responses were stored securely in Google Forms’ encrypted cloud storage, accessible only to the principal investigator. Incomplete or duplicate entries were automatically flagged and excluded through platform validation and manual screening.

### Data analysis

Data analysis was performed using GraphPad Prism version 10. Descriptive statistics, including means and standard deviations, were used to summarize attitude scores. Group comparisons were conducted using one-way ANOVA with post-hoc Tukey tests for pairwise comparisons. Pearson’s correlation coefficient was calculated to assess relationships between attitude scores and demographic variables. A *p*-value < 0.05 was considered statistically significant.

## Results

### Participant characteristics

A total of 166 students participated in the study, including 88 males (53.01%) and 78 females (46.99%), with a mean age of 22.78 ± 2.81 years. The majority of participants were single (93.97%) and lived in urban areas (92.77%). More than half (52.4%) resided in university dormitories, and 72.89% reported an average family financial status. The distribution of participants by field of study was as follows: 66 medical students (39.76%), 27 dental students (16.27%), 25 medical laboratory students (15.06%), 27 radiology students (16.27%), 10 anesthesiology students (6.02%), and 11 operating room students (6.63%). Table 1 summarizes the demographic characteristics of the participants.

**Table 1:** Demographic information of students participating in the study

Variable name	Status	*N* (%)
Gender	Male	88 (53.01%)
	Female	78 (46.99%)
Marital status	Single	156 (93.97%)
	Married	10 (6.03%)
Place of residence	City	154 (92.77%)
	Village	12 (7.22%)
Father’s education	Elementary, middle School	25 (15.06%)
	High School	34 (20.48%)
	Community Colleges	21 (12.65%)
	Bachelor’s Degree	42 (25.3%)
	Master’s Degree	34 (20.48%)
	Doctoral Degree or Ph.D	10 (6.02%)
Mother’s education	Elementary, middle School	65 (39.15%)
	High School	43 (25.9%)
	Community Colleges	11 (6.62%)
	Bachelor’s Degree	40 (24.09%)
	Master’s Degree	6 (3.61%)
	Doctoral Degree or Ph.D	1 (0.6%)
Student Housing Status	Dormitory	87 (52.4%)
	Non-Dormitory	79 (47.6%)
Financial status	Poor	35 (21.08%)
	Average	121 (72.89%)
	Good	10 (6.02%)
Distribution of participants by field of study	Medical students	66 (39.76%)
	Dental students	27 (16.27%)
	Medical laboratory students	25 (15.06%)
	Radiology students	27 (16.27%)
	Anesthesiology students	10 (6.02%)
	Operating room students	11 (6.63%)

### Attitude towards field of study

**Fig. 1:**
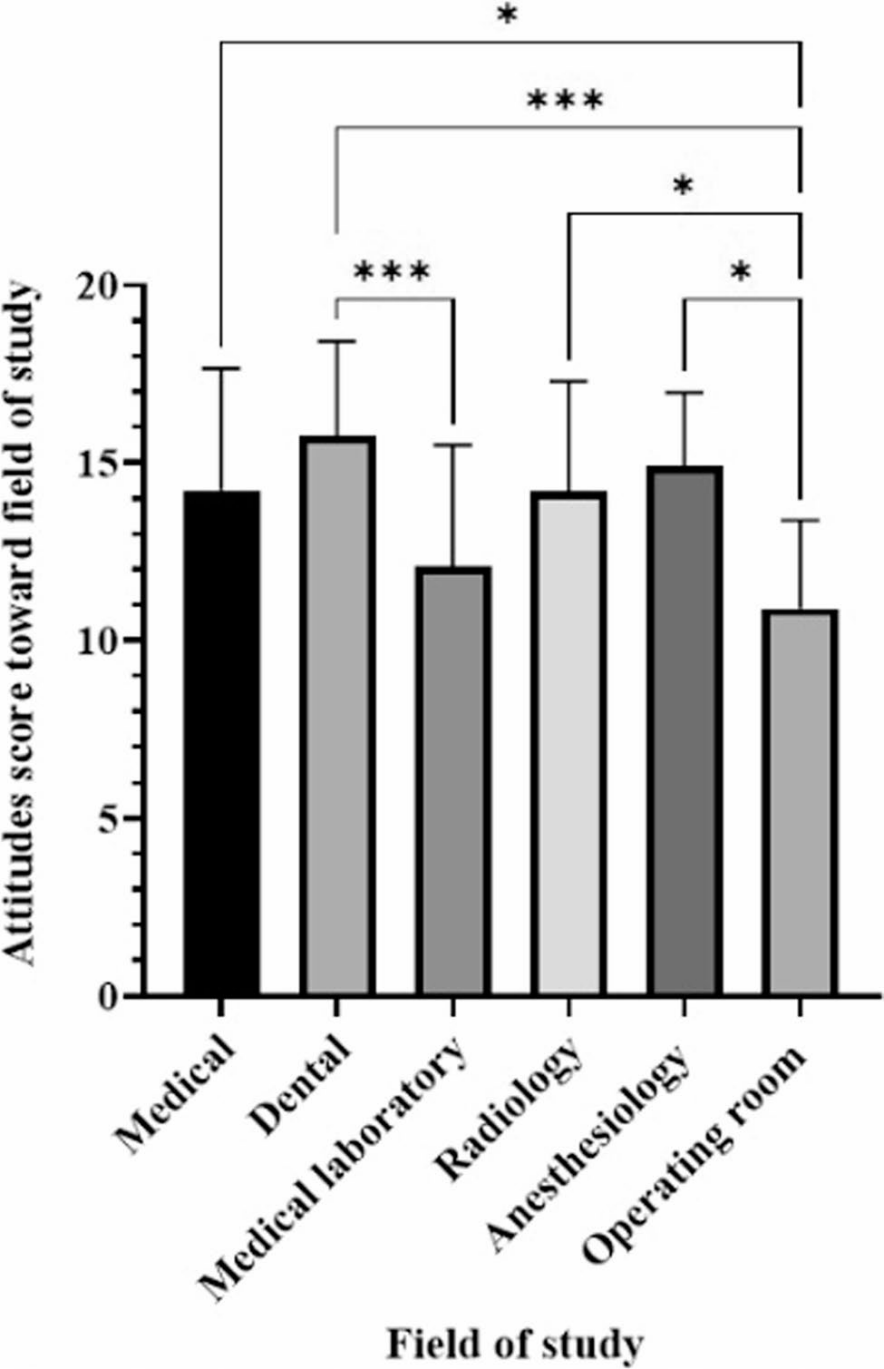
The mean score reflecting the attitude of medical, dental, and paramedical students towards their field of study is presented. Statistical significance is denoted as follows: *p ≤ 0.05, ***p < 0.001. Data are expressed as mean ± SD

Figure 1 shows the distribution of mean attitude scores toward students’ current field of study. Dental students reported the highest mean score (15.74 ± 2.68), while operating room students reported the lowest mean score (10.91 ± 2.46). Significant differences were found between medical students and operating room students (*p* < 0.05), as well as between dentistry students and both laboratory science and operating room students (*p* < 0.05).

**Fig. 2:**
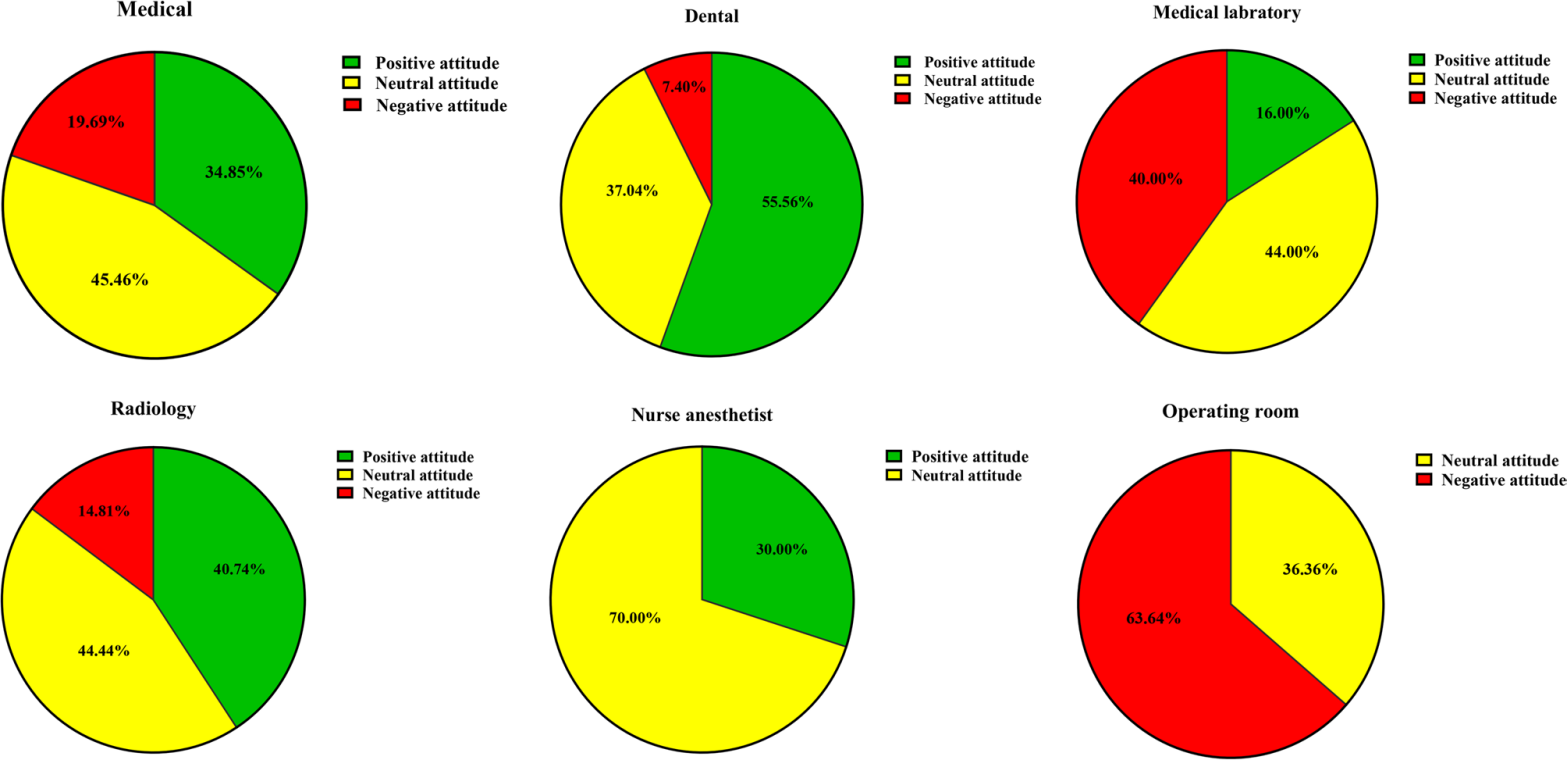
Distribution of positive, neutral, and negative attitudes among medical, dental, and paramedical students toward their fields of study

As shown in Table 2 and Fig. 2, attitude classifications using Bloom’s criteria indicated that 55.56% of dental students had positive attitudes, while the majority of students in other fields, including medicine, laboratory science, radiology, nurse anesthetist, and operating room, were classified as having neutral or negative attitudes toward their field of study.

**Table 2:** A breakdown of the number and percentage of medical, dental, and paramedical students exhibiting positive, neutral, and negative attitudes toward their respective fields of study

Field of study/Attitude to field of study	Medical	Dental	Medical laboratory	Radiology	Anesthesiology	Operating room
Positive	23 (34.85%)	15 (55.56%)	4 (16%)	11 (40.74%)	3 (30%)	0 (0%)
Neutral	30 (45.46%)	10 (37.04%)	11 (44%)	12 (44.44%)	7 (70%)	4 (36.36%)
Negative	13 (19.69%)	2 (7.4%)	10 (40%)	4 (14.81%)	0 (0%)	7 (63.64%)

### Attitudes toward future education and career

**Fig. 3:**
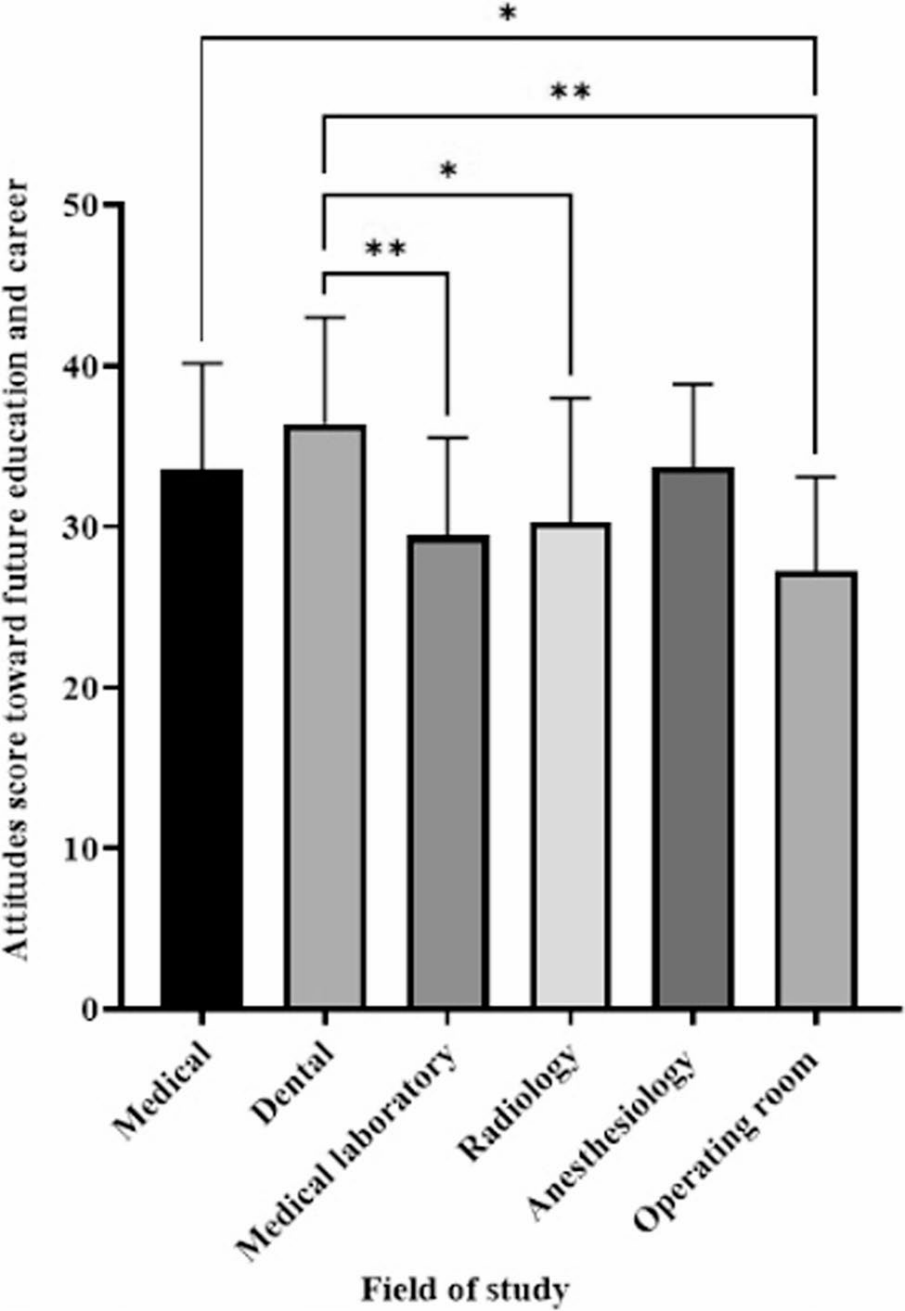
The average attitude score of medical, dental, and paramedical students regarding their future education and career is presented. Statistical significance is shown with (*p ≤ 0.05) and (**p ≤ 0.01). The data are expressed as mean ± SD

Mean scores for attitudes toward future education and career are presented in Fig. 3. Dental students again reported the highest mean score (36.41 ± 6.61), whereas students in the operating room reported the lowest mean score (27.27 ± 5.83). Medical students had significantly higher average scores compared to operating room students (*p* < 0.05), and dentistry students had significantly higher scores than laboratory science, radiology, and operating room students (*p* < 0.05).

**Fig. 4:**
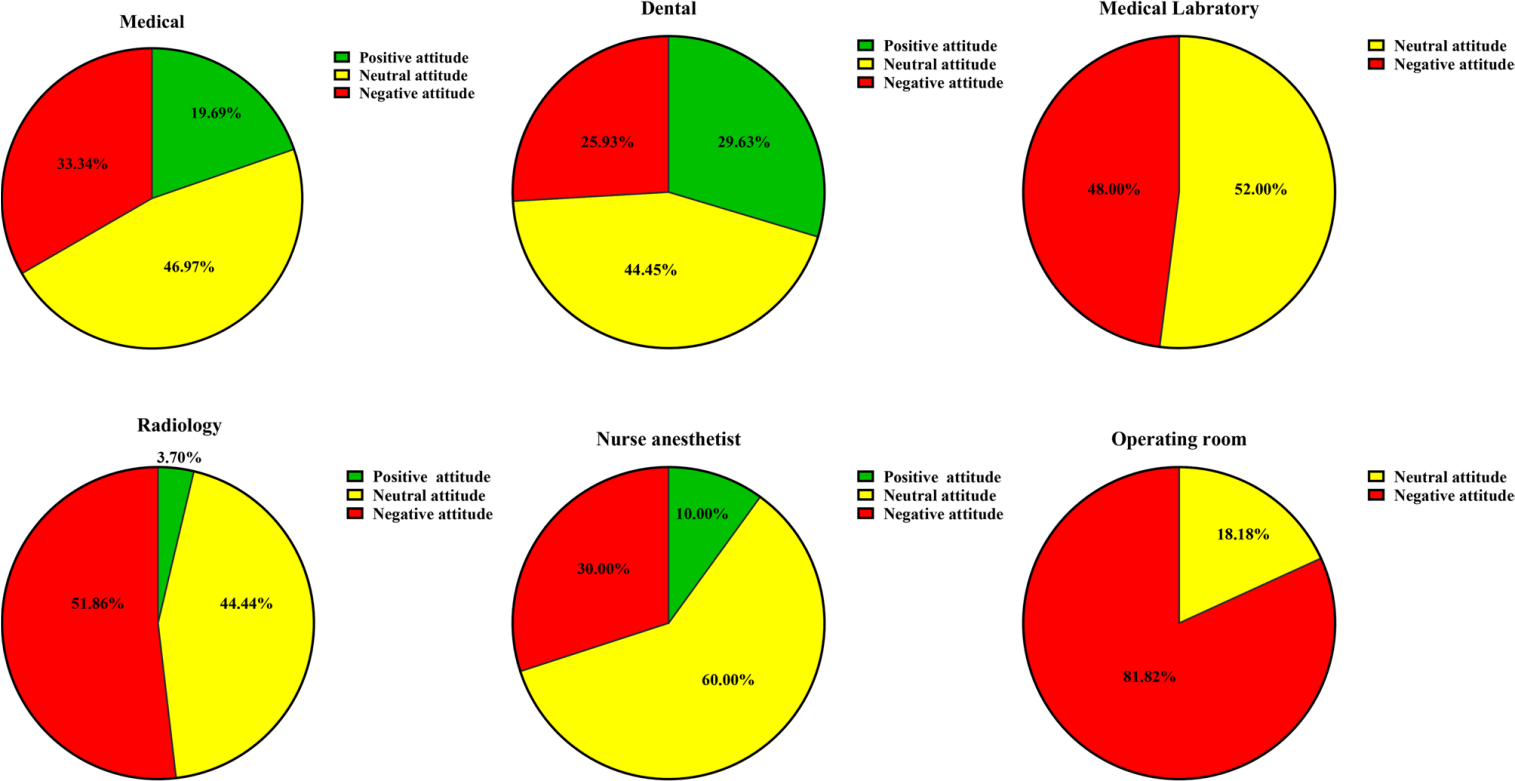
The distribution of positive, neutral, and negative attitudes among medical, dental, and paramedical students regarding their future education and career prospects

Table 3 and Fig. 4 summarize the distribution of attitude classifications toward future education and career. Most students across all fields were classified as having neutral or negative attitudes, including 100% of operating room and medical laboratory students.

**Table 3:** An analysis of the number and percentage of medical, dental, and paramedical students showing positive, neutral, and negative attitudes toward their future education and career

Field of study/Attitude to future education and career	Medical	Dental	Medical laboratory	Radiology	Anesthesiology	Operating room
Positive	13 (19.69%)	8 (29.62%)	0 (0%)	1 (3.7%)	1 (10%)	0 (0%)
Neutral	31 (46.97%)	12 (44.45%)	13 (52%)	12 (44.44%)	6 (60%)	2 (18.18%)
Negative	22 (33.34%)	7 (25.93%)	12 (48%)	14 (51.86%)	3 (30%)	9 (81.82%)

### Correlation with demographic variables

Pearson correlation coefficients indicated weak but statistically significant associations between attitude toward field of study and academic term (*r* = −0.17, *p* = 0.02) and gender (*r* = 0.15, *p* = 0.04). A similarly weak negative correlation was found between attitudes toward future education and career and academic term (*r* = − 0.15, *p* = 0.04). No statistically significant correlations were found with other demographic variables, including marital status, parental education (father’s and mother’s), housing status, and financial status.

### Item-level distribution of attitudes

In addition to overall attitude scores, item-level analysis was conducted to explore specific perceptions related to income, job stability, and employment opportunities across disciplines. In response to the statement “In my opinion, the income of this field is suitable,” Dentistry students reported the highest level of agreement (81.5%), indicating strong perceived financial viability within the field. In contrast, agreement levels were substantially lower among students in Radiology (29.6%), Operating Room (27.3%), Nurse Anesthetist (20.0%), and Laboratory Sciences (12.0%). These findings highlight a pronounced disparity in perceived income adequacy across disciplines, with paramedical students expressing notably lower levels of financial satisfaction compared to their dental counterparts.

For the statement “I am not worried about the career future in this field,” Dentistry students again demonstrated the highest level of agreement (48.1%), reflecting comparatively stronger confidence in future career stability. Conversely, agreement was substantially lower among students in Radiology (18.5%) and Laboratory Sciences (12.0%), underscoring significant apprehension about long-term job security within these fields.

Regarding the statement “The number of employment opportunities in this field within the country is appropriate,” the highest agreement was again observed among Dentistry students (48.1%), suggesting relatively optimistic views of domestic job availability. In contrast, agreement levels were markedly lower among students in Radiology (25.9%), Laboratory Sciences (8.0%), and Operating Room (9.1%), reflecting widespread concerns about employment saturation and potential misalignment between educational training and labor market demand.

A full breakdown of item-wise response distributions by discipline is provided in Supplementary Material 2.

## Discussion

This study investigated attitudes among medical, dental, and bachelor’s degree paramedical students toward their academic field, future education, and career. The findings revealed significant variations in student attitudes across disciplines, with dental students showing the most positive outlooks and operating room students the least. These differences highlight the role of both intrinsic (e.g., job satisfaction, professional autonomy) and extrinsic factors (e.g., financial incentives, career opportunities) in shaping students’ perceptions [19].

### Field-specific variations in student attitudes

Dental students reported the highest mean attitude scores toward their field of study and future career, whereas operating room students consistently showed the lowest scores. This pattern is further supported by item-level responses. For example, 81.5% of dental students agreed or strongly agreed that the income in their field is suitable, compared to only 27.3% in operating room and 12.0% in laboratory sciences. Similarly, when asked whether they were worried about their career future, 48.1% of dental students expressed confidence, while agreement dropped to 18.5% and 12.0% in radiology and laboratory sciences, respectively.

These discipline-specific differences align with earlier studies showing that medical and dental students report higher levels of satisfaction and professional confidence than paramedical students, who often experience dissatisfaction with income, job security, and professional recognition [20, 21]. This suggests a potential mismatch between student expectations and structural realities in healthcare job markets.

Moreover, similar patterns have been documented in Iranian healthcare education, where paramedical students consistently express lower satisfaction and diminished optimism toward their future careers [20, 22]. Negative attitudes formed at this stage may serve as early indicators of broader workforce disengagement.

### Distribution patterns of attitudinal categories

Analysis based on Bloom’s cut-off points revealed that the majority of students across all fields were classified as having neutral or negative attitudes toward both their current academic field and their future careers. Specifically, 100% of students in operating room and medical laboratory programs fell into the neutral or negative categories. Similarly, more than 90% of radiology and nurse anesthetist students and over 80% of medical students were also categorized in the lower attitude tiers. Notably, even among dental students—who had the highest mean scores—70.37% expressed a neutral or negative attitude toward their future education and career prospects.

These patterns underscore the widespread presence of attitudinal disengagement across healthcare education disciplines. This disengagement is particularly evident in perceptions of employment opportunities. While 48.1% of dental students believed that sufficient job opportunities exist in their field, agreement rates fell sharply to 25.9% in radiology, 9.1% in operating room, and just 8.0% in laboratory sciences. These data confirm the existence of structural disparities that affect students’ outlooks, especially in oversaturated paramedical programs (see Supplementary Material 2).

These findings are consistent with earlier research in which dissatisfaction with education and career prospects has been reported among paramedical students in Iran [22]. A key contributing factor may be the growing gap between student enrollment and available job market opportunities, particularly in paramedical fields. This mismatch is further compounded by workforce oversaturation, reduced financial incentives, and broader economic instability in Iran, including rising inflation and depreciation of the national currency [23]. These structural barriers, combined with limited opportunities for professional advancement, may contribute to students’ loss of motivation and increasing disinterest in pursuing careers within their field of study. A systematic review of transition-to-practice programs for allied health graduates in the UK and Australia revealed a similar pattern: a persistent misalignment between student expectations and workplace realities, especially in terms of job readiness, support mechanisms, and long-term career development [24]. These systemic gaps may contribute to the formation of neutral and negative attitudes toward future careers even before graduation.

Similar patterns have been reported, where inadequate employment prospects and salary disparities have driven an increasing number of healthcare graduates to seek opportunities abroad [25, 26]. Addressing these concerns through strategic workforce planning, expansion of career development programs, and the implementation of financial incentive structures may be essential in improving students’ professional outlook and reversing negative attitude trends within the healthcare education system.

### Influence of demographic factors on attitude

Weak but statistically significant correlations were observed between academic term and student attitudes, suggesting a gradual decline in optimism as students’ progress through their studies and encounter academic stress, systemic challenges, and limited practical exposure [27]. A small gender-related difference was also detected, though its predictive value appears limited. These findings align with previous studies reporting that demographic variables such as gender and age exert only a modest influence on career attitudes among healthcare students [28–30].

Nevertheless, other research indicates that personal and contextual factors—such as age, gender, and lived experiences—may substantially affect career choices and specialty preferences [31, 32]. For example, studies on nursing students have shown that gender role stereotypes and patriarchal family environments influence professional satisfaction and specialty selection, with male students in more patriarchal contexts exhibiting stronger adherence to traditional roles. Likewise, younger students, those with stronger academic performance, and individuals who voluntarily select their major are more likely to report positive career attitudes [32]. Taken together, these findings suggest that while demographic factors alone may play a modest role, their impact is often mediated by broader cultural, social, and personal contexts that ultimately shape students’ professional outlook.

### Broader structural and economic implications

Iran’s healthcare system provides a striking case of how negative professional attitudes may lead to larger systemic consequences [33–36]. Over the past two decades, the country has witnessed an intensifying “brain drain” among healthcare professionals. A 2017 WHO report ranked Iran seventh globally in physician emigration, with approximately 13,000 Iranian doctors having relocated to 35 OECD countries—a 40% increase compared to 2000 [37]. By 2022, over 50% of Iranian medical personnel reported intentions to emigrate [38, 39].

This trend has profound economic consequences. Estimates indicate that educating a general practitioner costs around $57,000, while each emigrated physician represents a potential loss of up to $450,000 in long-term economic contributions [40]. These shortages exacerbate systemic issues, including increased wait times, reduced quality of care, and rising public dissatisfaction [41–43].

Our findings suggest that such macro-level outcomes may be rooted in micro-level educational experiences. The high prevalence of neutral and negative attitudes—especially among paramedical students—reflects early professional disengagement that, if left unaddressed, may evolve into emigration intentions and eventual workforce attrition.

### Educational and policy implications

These findings hold important implications for educational policymakers and healthcare workforce planners. The high prevalence of negative and neutral attitudes—particularly among paramedical students—signals a potential crisis in professional engagement and long-term retention. Academic institutions should implement early-stage career counseling, field-specific mentorship programs, and realistic job market orientation sessions to help align student expectations with workforce realities.

In addition, admission policies should be reviewed to ensure alignment between educational capacity and labor market demands. Over-admission in certain paramedical fields, without corresponding employment opportunities, may exacerbate frustration and contribute to negative attitudes. Governmental bodies should also consider providing targeted financial incentives, job security schemes, and structured career pathways to reduce attrition and brain drain [44–48].

### Directions for future research

Future studies should explore the underlying causes of student attitudes using qualitative methods such as interviews or focus groups, particularly in fields with consistently negative responses (e.g., operating room and radiology). Longitudinal studies could also help track the evolution of attitudes over the course of students’ education and early career stages. Investigating institutional culture, faculty support, and perceptions of professional value could further illuminate modifiable factors influencing attitudes.

### Limitations

This study has several limitations. First, it was conducted at a single institution, which may limit generalizability to other academic settings. Second, the relatively small sample size restricts the statistical power for subgroup analyses. Third, the cross-sectional design precludes causal interpretations. Nonetheless, the study provides valuable insights into patterns of student attitudes and highlights areas in need of further investigation.

## Conclusion

This study highlights significant differences in students’ attitudes toward their academic fields and future careers across medical, dental, and paramedical disciplines. Dental students had the most positive attitudes, while operating room students showed the most negative attitudes, particularly regarding career prospects. The study reveals systemic issues in healthcare education, such as misalignment between academic training and labor market realities, limited career visibility, and financial concerns, especially among paramedical students.

To address these challenges, the study recommends early career counseling, mentorship programs, clearer career pathways, and reassessing enrollment strategies. These interventions aim to align students’ academic experiences with labor market demands, particularly for paramedical students, and improve their career confidence and satisfaction. By implementing these reforms, the study contributes to enhancing student engagement and long-term workforce stability in healthcare education.

## Supplementary Information

Supplementary Material 1

Supplementary Material 2

## Data Availability

The datasets utilized and/or analyzed in this study are available from the corresponding author upon request.

## Abbreviations

SD: Standard deviations
OECD: Organization for Economic Cooperation and Development
Ph.D: Philosophiae Doctor
