# Supplementary material for: Disparities in attitudes toward field of study and future career among students at Yasuj University of medical sciences
Source: BMC Med Educ. 2025 Dec 20;26:136. doi: 10.1186/s12909-025-08087-6 (PMC12837015; doi:10.1186/s12909-025-08087-6)
Supplement: Supplementary file 1 — Supplementary Material 1 [file 12909_2025_8087_MOESM1_ESM.docx]

**Questionnaire of students' attitude towards the field of study and future education and career**

*With full knowledge of the above, I agree to participate in the aforementioned research as a study subject.

I agree 🗆

* Field of study?

Medicine 🗆

Dentistry 🗆

Laboratory science 🗆

Operating room 🗆

Nurse anesthetist 🗆

Radiology 🗆

2- Academic semester?

* Age?

* Marital status?

Single 🗆

Married 🗆

* Place of residence?

City 🗆

Village 🗆

* Student Housing Status?

a dormitory 🗆

non-dormitory 🗆

* Family financial level:

good 🗆

average 🗆

bad 🗆

* Father's education

Elementary, middle School 🗆

High School 🗆

Community Colleges 🗆

Bachelor's Degree 🗆

Master's Degree 🗆

Doctoral Degree or Ph.D 🗆

* Mother's education

Elementary, middle School 🗆

High School 🗆

Community Colleges 🗆

Bachelor's Degree 🗆

Master's Degree 🗆

Doctoral Degree or Ph.D 🗆

1- I am satisfied with choosing this field.

Totally agree 🗆

Agree 🗆

Neutral/no opinion 🗆

Disagree🗆

Totally disagree🗆

2- The goals of the courses offered in this field are tailored to the individual needs and expectations of students.

Totally agree 🗆

Agree 🗆

Neutral/no opinion 🗆

Disagree🗆

Totally disagree🗆

3- After studying this field, I became more interested in it.

Totally agree 🗆

Agree 🗆

Neutral/no opinion 🗆

Disagree🗆

Totally disagree🗆

4- In my opinion, the society in which I live has a positive attitude towards this field.

Totally agree 🗆

Agree 🗆

Neutral/no opinion 🗆

Disagree🗆

Totally disagree🗆

5- In my opinion, this field becomes more valuable in higher grades.

Totally agree 🗆

Agree 🗆

Neutral/no opinion 🗆

Disagree🗆

Totally disagree🗆

6- The opinion of students of other fields is suitable for my field.

Totally agree 🗆

Agree 🗆

Neutral/no opinion 🗆

Disagree🗆

Totally disagree🗆

7- I hope to continue studying in this field.

Totally agree 🗆

Agree 🗆

Neutral/no opinion 🗆

Disagree🗆

Totally disagree🗆

8- Professors of this field encourage students to find a suitable job.

Totally agree 🗆

Agree 🗆

Neutral/no opinion 🗆

Disagree🗆

Totally disagree🗆

9- In my opinion, the income of this field is suitable.

Totally agree 🗆

Agree 🗆

Neutral/no opinion 🗆

Disagree🗆

Totally disagree🗆

10- I am not worried about the career future in this field.

Totally agree 🗆

Agree 🗆

Neutral/no opinion 🗆

Disagree🗆

Totally disagree🗆

11- In my opinion, there is a proper counseling system in the university in the field of future career.

Totally agree 🗆

Agree 🗆

Neutral/no opinion 🗆

Disagree🗆

Totally disagree🗆

12- I like working in my field of study.

Totally agree 🗆

Agree 🗆

Neutral/no opinion 🗆

Disagree🗆

Totally disagree🗆

13- In my opinion, the employment situation in this field in Iran is more suitable for higher levels.

Totally agree 🗆

Agree 🗆

Neutral/no opinion 🗆

Disagree🗆

Totally disagree🗆

14- In my opinion, the number of employment opportunities in this field within the country is appropriate.

Totally agree 🗆

Agree 🗆

Neutral/no opinion 🗆

Disagree🗆

Totally disagree🗆
