# Supplementary material for: Disparities in attitudes toward field of study and future career among students at Yasuj University of medical sciences
Source: BMC Med Educ. 2025 Dec 20;26:136. doi: 10.1186/s12909-025-08087-6 (PMC12837015; doi:10.1186/s12909-025-08087-6)
Supplement: Supplementary file 2 — Supplementary Material 2 [file 12909_2025_8087_MOESM2_ESM.docx]

# Appendix A – Distribution of Likert-Scale Responses by Field of Study

This appendix provides a detailed breakdown of students’ responses to Likert-scale attitude items across all academic disciplines. Each item reflects a specific construct related to students' attitude towards the field of study and future education and career. Responses are presented as percentages by field of study.

Likert Scale:

- 1 = Strongly Disagree
- 2 = Disagree
- 3 = Neutral
- 4 = Agree
- 5 = Strongly Agree

| Item | Field of Study | Strongly Disagree (1) | Disagree (2) | Neutral (3) | Agree (4) | Strongly Agree (5) |
| --- | --- | --- | --- | --- | --- | --- |
| 1- I am satisfied with choosing this field. | Medicine | 1.5 | 6.1 | 15.2 | 37.9 | 39.4 |
| 1- I am satisfied with choosing this field. | Dentistry | 0.0 | 0.0 | 11.1 | 48.1 | 40.7 |
| 1- I am satisfied with choosing this field. | Laboratory Science | 16.0 | 0.0 | 28.0 | 44.0 | 12.0 |
| 1- I am satisfied with choosing this field. | Radiology | 3.7 | 14.8 | 14.8 | 33.3 | 33.3 |
| 1- I am satisfied with choosing this field. | Nurse Anesthetist | 0.0 | 0.0 | 0.0 | 80.0 | 20.0 |
| 1- I am satisfied with choosing this field. | Operating Room | 0.0 | 27.3 | 54.5 | 9.1 | 9.1 |
| 2- The goals of the courses offered in this field are tailored to the individual needs and expectations of students. | Medicine | 9.1 | 27.3 | 24.2 | 33.3 | 6.1 |
| 2- The goals of the courses offered in this field are tailored to the individual needs and expectations of students. | Dentistry | 0.0 | 29.6 | 22.2 | 40.7 | 7.4 |
| 2- The goals of the courses offered in this field are tailored to the individual needs and expectations of students. | Laboratory Science | 0.0 | 28.0 | 36.0 | 36.0 | 0.0 |
| 2- The goals of the courses offered in this field are tailored to the individual needs and expectations of students. | Radiology | 7.4 | 40.7 | 14.8 | 37.0 | 0.0 |
| 2- The goals of the courses offered in this field are tailored to the individual needs and expectations of students. | Nurse Anesthetist | 10.0 | 30.0 | 20.0 | 30.0 | 10.0 |
| 2- The goals of the courses offered in this field are tailored to the individual needs and expectations of students. | Operating Room | 9.1 | 54.5 | 18.2 | 18.2 | 0.0 |
| 3- After studying this field, I became more interested in it. | Medicine | 7.6 | 18.2 | 15.2 | 34.8 | 24.2 |
| 3- After studying this field, I became more interested in it. | Dentistry | 0.0 | 11.1 | 11.1 | 51.9 | 25.9 |
| 3- After studying this field, I became more interested in it. | Laboratory Science | 16.0 | 20.0 | 16.0 | 32.0 | 16.0 |
| 3- After studying this field, I became more interested in it. | Radiology | 3.7 | 22.2 | 11.1 | 33.3 | 29.6 |
| 3- After studying this field, I became more interested in it. | Nurse Anesthetist | 0.0 | 0.0 | 20.0 | 50.0 | 30.0 |
| 3- After studying this field, I became more interested in it. | Operating Room | 0.0 | 27.3 | 9.1 | 63.6 | 0.0 |
| 4- In my opinion, the society in which I live has a positive attitude towards this field. | Medicine | 7.6 | 13.6 | 16.7 | 31.8 | 30.3 |
| 4- In my opinion, the society in which I live has a positive attitude towards this field. | Dentistry | 0.0 | 7.4 | 3.7 | 44.4 | 44.4 |
| 4- In my opinion, the society in which I live has a positive attitude towards this field. | Laboratory Science | 20.0 | 28.0 | 32.0 | 20.0 | 0.0 |
| 4- In my opinion, the society in which I live has a positive attitude towards this field. | Radiology | 3.7 | 3.7 | 11.1 | 55.6 | 25.9 |
| 4- In my opinion, the society in which I live has a positive attitude towards this field. | Nurse Anesthetist | 0.0 | 20.0 | 20.0 | 40.0 | 20.0 |
| 4- In my opinion, the society in which I live has a positive attitude towards this field. | Operating Room | 18.2 | 54.5 | 27.3 | 0.0 | 0.0 |
| 5- In my opinion, this field becomes more valuable in higher grades. | Medicine | 1.5 | 4.5 | 6.1 | 42.4 | 45.5 |
| 5- In my opinion, this field becomes more valuable in higher grades. | Dentistry | 0.0 | 11.1 | 25.9 | 33.3 | 29.6 |
| 5- In my opinion, this field becomes more valuable in higher grades. | Laboratory Science | 0.0 | 0.0 | 12.0 | 44.0 | 44.0 |
| 5- In my opinion, this field becomes more valuable in higher grades. | Radiology | 7.4 | 11.1 | 33.3 | 25.9 | 22.2 |
| 5- In my opinion, this field becomes more valuable in higher grades. | Nurse Anesthetist | 0.0 | 10.0 | 20.0 | 50.0 | 20.0 |
| 5- In my opinion, this field becomes more valuable in higher grades. | Operating Room | 0.0 | 27.3 | 9.1 | 45.5 | 18.2 |
| 6- The opinion of students of other fields is suitable for my field. | Medicine | 3.0 | 7.6 | 15.2 | 42.4 | 31.8 |
| 6- The opinion of students of other fields is suitable for my field. | Dentistry | 0.0 | 3.7 | 7.4 | 48.1 | 40.7 |
| 6- The opinion of students of other fields is suitable for my field. | Laboratory Science | 8.0 | 20.0 | 52.0 | 16.0 | 4.0 |
| 6- The opinion of students of other fields is suitable for my field. | Radiology | 0.0 | 0.0 | 11.1 | 77.8 | 11.1 |
| 6- The opinion of students of other fields is suitable for my field. | Nurse Anesthetist | 0.0 | 0.0 | 40.0 | 60.0 | 0.0 |
| 6- The opinion of students of other fields is suitable for my field. | Operating Room | 0.0 | 36.4 | 45.5 | 18.2 | 0.0 |
| 7- I hope to continue studying in this field. | Medicine | 6.1 | 1.5 | 16.7 | 36.4 | 39.4 |
| 7- I hope to continue studying in this field. | Dentistry | 0.0 | 11.1 | 7.4 | 55.6 | 25.9 |
| 7- I hope to continue studying in this field. | Laboratory Science | 28.0 | 4.0 | 12.0 | 40.0 | 16.0 |
| 7- I hope to continue studying in this field. | Radiology | 14.8 | 7.4 | 29.6 | 29.6 | 18.5 |
| 7- I hope to continue studying in this field. | Nurse Anesthetist | 0.0 | 0.0 | 20.0 | 60.0 | 20.0 |
| 7- I hope to continue studying in this field. | Operating Room | 0.0 | 45.5 | 18.2 | 36.4 | 0.0 |
| 8- Professors of this field encourage students to find a suitable job. | Medicine | 24.2 | 33.3 | 25.8 | 12.1 | 4.5 |
| 8- Professors of this field encourage students to find a suitable job. | Dentistry | 7.4 | 11.1 | 37.0 | 37.0 | 7.4 |
| 8- Professors of this field encourage students to find a suitable job. | Laboratory Science | 20.0 | 12.0 | 36.0 | 32.0 | 0.0 |
| 8- Professors of this field encourage students to find a suitable job. | Radiology | 22.2 | 14.8 | 29.6 | 22.2 | 11.1 |
| 8- Professors of this field encourage students to find a suitable job. | Nurse Anesthetist | 30.0 | 20.0 | 10.0 | 20.0 | 20.0 |
| 8- Professors of this field encourage students to find a suitable job. | Operating Room | 18.2 | 36.4 | 36.4 | 9.1 | 0.0 |
| 9- In my opinion, the income of this field is suitable. | Medicine | 13.6 | 27.3 | 19.7 | 34.8 | 4.5 |
| 9- In my opinion, the income of this field is suitable. | Dentistry | 0.0 | 0.0 | 18.5 | 55.6 | 25.9 |
| 9- In my opinion, the income of this field is suitable. | Laboratory Science | 32.0 | 40.0 | 16.0 | 12.0 | 0.0 |
| 9- In my opinion, the income of this field is suitable. | Radiology | 22.2 | 33.3 | 14.8 | 22.2 | 7.4 |
| 9- In my opinion, the income of this field is suitable. | Nurse Anesthetist | 40.0 | 10.0 | 30.0 | 20.0 | 0.0 |
| 9- In my opinion, the income of this field is suitable. | Operating Room | 36.4 | 27.3 | 9.1 | 27.3 | 0.0 |
| 10- I am not worried about the career future in this field. | Medicine | 24.2 | 25.8 | 15.2 | 30.3 | 4.5 |
| 10- I am not worried about the career future in this field. | Dentistry | 0.0 | 37.0 | 14.8 | 25.9 | 22.2 |
| 10- I am not worried about the career future in this field. | Laboratory Science | 28.0 | 36.0 | 24.0 | 8.0 | 4.0 |
| 10- I am not worried about the career future in this field. | Radiology | 29.6 | 40.7 | 11.1 | 11.1 | 7.4 |
| 10- I am not worried about the career future in this field. | Nurse Anesthetist | 0.0 | 50.0 | 20.0 | 30.0 | 0.0 |
| 10- I am not worried about the career future in this field. | Operating Room | 27.3 | 18.2 | 9.1 | 27.3 | 18.2 |
| 11- In my opinion, there is a proper counseling system in the university in the field of future career. | Medicine | 37.9 | 30.3 | 24.2 | 6.1 | 1.5 |
| 11- In my opinion, there is a proper counseling system in the university in the field of future career. | Dentistry | 14.8 | 33.3 | 44.4 | 0.0 | 7.4 |
| 11- In my opinion, there is a proper counseling system in the university in the field of future career. | Laboratory Science | 28.0 | 20.0 | 48.0 | 4.0 | 0.0 |
| 11- In my opinion, there is a proper counseling system in the university in the field of future career. | Radiology | 29.6 | 33.3 | 22.2 | 7.4 | 7.4 |
| 11- In my opinion, there is a proper counseling system in the university in the field of future career. | Nurse Anesthetist | 20.0 | 10.0 | 30.0 | 30.0 | 10.0 |
| 11- In my opinion, there is a proper counseling system in the university in the field of future career. | Operating Room | 36.4 | 18.2 | 45.5 | 0.0 | 0.0 |
| 12- I like working in my field of study. | Medicine | 0.0 | 4.5 | 0.0 | 48.5 | 47.0 |
| 12- I like working in my field of study. | Dentistry | 0.0 | 0.0 | 18.5 | 48.1 | 33.3 |
| 12- I like working in my field of study. | Laboratory Science | 8.0 | 16.0 | 8.0 | 52.0 | 16.0 |
| 12- I like working in my field of study. | Radiology | 7.4 | 7.4 | 18.5 | 33.3 | 33.3 |
| 12- I like working in my field of study. | Nurse Anesthetist | 0.0 | 0.0 | 0.0 | 50.0 | 50.0 |
| 12- I like working in my field of study. | Operating Room | 0.0 | 9.1 | 27.3 | 63.6 | 0.0 |
| 13- In my opinion, the employment situation in this field in Iran is more suitable for higher levels. | Medicine | 3.0 | 6.1 | 7.6 | 40.9 | 42.4 |
| 13- In my opinion, the employment situation in this field in Iran is more suitable for higher levels. | Dentistry | 0.0 | 14.8 | 18.5 | 51.9 | 14.8 |
| 13- In my opinion, the employment situation in this field in Iran is more suitable for higher levels. | Laboratory Science | 4.0 | 12.0 | 8.0 | 52.0 | 24.0 |
| 13- In my opinion, the employment situation in this field in Iran is more suitable for higher levels. | Radiology | 3.7 | 11.1 | 44.4 | 29.6 | 11.1 |
| 13- In my opinion, the employment situation in this field in Iran is more suitable for higher levels. | Nurse Anesthetist | 0.0 | 10.0 | 20.0 | 60.0 | 10.0 |
| 13- In my opinion, the employment situation in this field in Iran is more suitable for higher levels. | Operating Room | 9.1 | 27.3 | 9.1 | 45.5 | 9.1 |
| 14- In my opinion, the number of employment opportunities in this field within the country is appropriate. | Medicine | 16.7 | 19.7 | 33.3 | 22.7 | 7.6 |
| 14- In my opinion, the number of employment opportunities in this field within the country is appropriate. | Dentistry | 0.0 | 29.6 | 22.2 | 29.6 | 18.5 |
| 14- In my opinion, the number of employment opportunities in this field within the country is appropriate. | Laboratory Science | 16.0 | 36.0 | 40.0 | 8.0 | 0.0 |
| 14- In my opinion, the number of employment opportunities in this field within the country is appropriate. | Radiology | 22.2 | 40.7 | 11.1 | 22.2 | 3.7 |
| 14- In my opinion, the number of employment opportunities in this field within the country is appropriate. | Nurse Anesthetist | 0.0 | 20.0 | 40.0 | 40.0 | 0.0 |
| 14- In my opinion, the number of employment opportunities in this field within the country is appropriate. | Operating Room | 54.5 | 36.4 | 0.0 | 9.1 | 0.0 |
